# Supplementary material for: Improving Nutrient Use Efficiency of Rice Under Alternative Wetting and Drying Irrigation Combined with Slow-Release Nitrogen Fertilization
Source: Plants (Basel). 2025 May 20;14(10):1530. doi: 10.3390/plants14101530 (PMC12114806; doi:10.3390/plants14101530)
Supplement: Supplementary file 1 [file plants-14-01530-s001.zip › plants-3627689-supplementary.pdf]

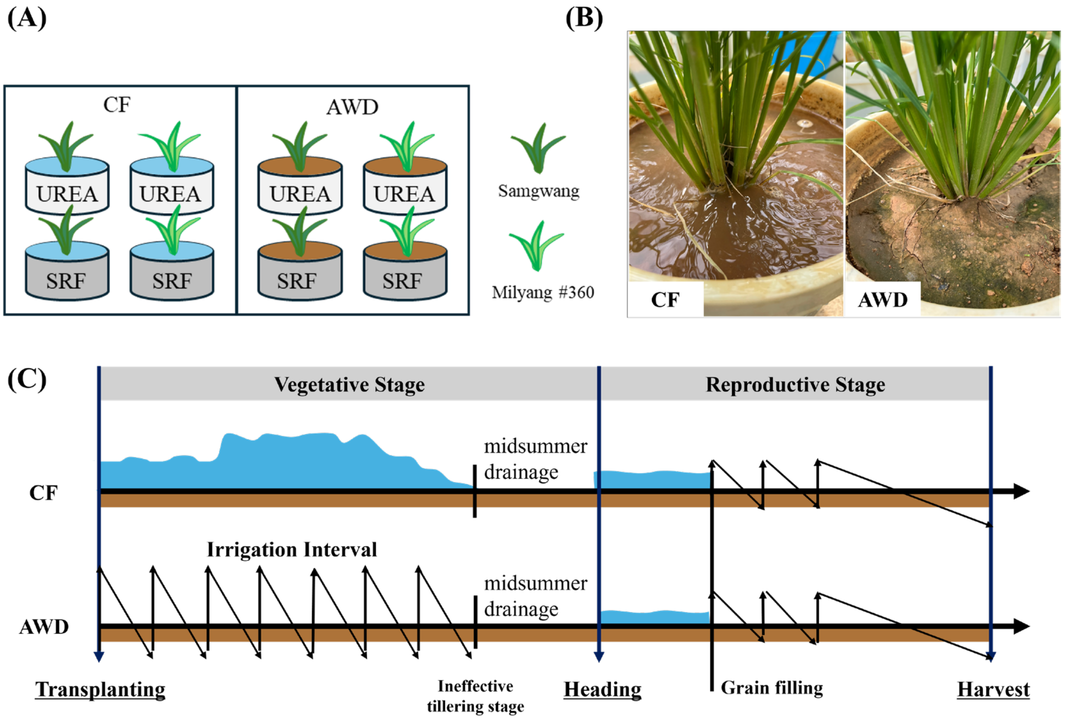


**Supplementary Figure S1.** Nitrogen fertilization and water management. (**A**) Pot arrangement, (**B**) Continuous flooding (CF) and alternate wetting and drying (AWD), and (**C**) After management throughout the entire growing season. Nitrogen was supplied either as urea or slow-release fertilizer (SRF)

**Supplementary Table S1.** qRT-PCR primer sequences and PCR conditions.

| Function | Gene Symbol | Tissue | Gene ID | Primer Sequence (5′→3′) | Tm (°C) | Size (bp) |
|---|---|---|---|---|---|---|
| House keeping | *OsACT-1* | | *Os03g0718100* | (FW) TGTATGCCAGTGGTCGTACC (RV) CCAGCAAGGTCGAGACGA A | | 186 |
| Ammonium uptake | *OsAMT1.1* | Root | *Os04g0509600* | (FW) ACGTCATCCAGATCCTGGT C (RV) AGACTTGTCGTGCTCGTCCT | 59 | 182 |
| | *OsAMT2.1* | Root | *Os05g0468700* | (FW) GGTGCTCTTCCAGTTCGAGT (RV) GACGGTGTAGGAGAGGAG GA | 59 | 127 |
| Nitrate uptake | *OsNRT1.1b* | Root | *Os10t0554200-01* | (FW) GTCACCATCGTCCACAAGG | 56 | 135 |

| | | | | | | |
|---|---|---|---|---|---|---|
| | | | | (RV) GAAGAGGACGAGGTTGATGG | | |
| | *OsNRT2.1* | Root | *Os02g0112100* | (FW) TACGCCGTCACCAACTACC (RV) GGTCGAAGCGATCGTAGAAG | 56 | 115 |
| Nitrogen assimilation | *OsNIR* | Leaf blade, root | *Os01t0357100-01* | (FW) ATCAACGACCTCGCGTACAT (RV) GAGAACGGCCTTGCACAC | 56 | 159 |
| | *OsNR* | Leaf blade, root | *Os02t0770800-01* | (FW) AGGGTAGAGGTGACCCTGGA (RV) GCTGGGTGTTGAGGGACTC | 56 | 187 |
| | *OsGs1.1* | Leaf blade | *Os02t0735200-01* | (FW) CTGTGGTATCGGTGCTGACA (RV) ATGACCTCGCCGTTGATTC | 56 | 108 |
| | *OsGs1.2* | Root | *Os03g0223400* | (FW) ACGGAGAAGGAGGGCAAG (RV) CCACAGCAGCGTGGTCTC | 59 | 102 |
| | *OsFd-GOGAT* | Leaf blade | *Os07t0658400-02* | (FW) ACCCTATCAAGTCCTGTGCTT (RV) AGCAGCATCAGCTTCATCAC | 56 | 162 |
| | *OsNADH-GOGAT1* | Root | *Os01t0681900-01* | (FW) GGCCTGGTCGATTTTATGTG (RV) GTGCCTTCAATGCTTCATCA | 53 | 174 |
| | *OsAS1* | Root | *Os03t0291500-01* | (FW) CTGAGCTCCATTCCTTCGTC (RV) CGTCTCGTCGTGGTAGATGA | 60 | 158 |
| | *OsAS2* | Leaf blade | *Os06t0265000-01* | (FW) AGGTGGCCTAAGGAGATGGT (RV) TCCAGTCCACCAGACAAGAG | 50 | 159 |
| | *OsAAT1* | Leaf blade | *Os02t0797500-01* | (FW) GGTGAAGAGCCAGCTGAAAC (RV) CACTGTCGTCCTTTGCAGAC | 56 | 197 |
| | *OsAAT2* | Root | *Os01t0760600-01* | (FW) CCCGTCACGTGTTAAGGAG (RV) CCTCCCACCCTTAAAGAACC | 56.9 | 159 |
